# The Influence of Patient and System Factors on the Radiotherapy Treatment Time in the Treatment of Non-metastatic Cervical Cancer Patients in a Rural and Resource-Lean State’s Safety-Net Hospital: Benefits of Strategic Planning

**DOI:** 10.7759/cureus.35954

**Published:** 2023-03-09

**Authors:** Srinivasan Vijayakumar, Mary R Nittala, William N Duggar, Maurice King, Seth T. Lirette, Claus Chunli Yang, Eswar Mundra, William C Woods, Jeremy Otts, Michael Doherty, Paige Panter, Candace Howard, Mildred Ridgway, Robert Allbright

**Affiliations:** 1 Radiation Oncology, University of Mississippi Medical Center, Jackson, USA; 2 Data Science, University of Mississippi Medical Center, Jackson, USA; 3 Radiology, University of Mississippi Medical Center, Jackson, USA; 4 Anesthesiology, University of Mississippi Medical Center, Jackson, USA; 5 Obstetrics and Gynecology, University of Mississippi Medical Center, Jackson, USA

**Keywords:** radiotherapy treatment time, elapsed days, treatment time, mri-guided brachytherapy, uterine cervical cancer

## Abstract

Objective

To decrease radiotherapy treatment time (RTT), measured from the day of initiation of radiotherapy to the day of its completion, specific strategies were initiated in early 2020 in the only academic safety-net medical center in a rural, resource-lean state. The factors that can succeed and those that need further improvements were analyzed in this initial assessment phase of our efforts to shorten the RTT.

Methods

This is an analysis of 28 cervix cancer patients treated with magnetic resonance imaging (MRI)-guided brachytherapy (February 2020-November 2021). The relationship between independent and dependent variable were analyzed by simple linear regression, and p-values ≤ 0.05 were considered statistically significant. SPSS software version 28.0 (IBM, Armonk, NY, USA) was used for statistical analysis.

Results

Two RTT groups (≤ 60 (32.1%) *vs*. > 60 days {67.9%}) with median RTT of 68 days (range, 51 to 106 days) were analyzed. Caucasians represented 66.7% of the RTT ≤ 60 days group. Four ‘issues’ were identified that increased the RTT: non-compliance, learning curve (early days of implementation of MRI-guided brachytherapy in the department), stage IV comorbidities, and with more than one issue mentioned; 77.8% with no issues had ≤ 60 days RTT *vs*. 26.3% for the > 60 days group. The breakdown of the no-issues factor by calendar year showed the RTT of ≤ 60 days was achieved higher in 2021 (85.7% *vs.* 20.0%; p=0.023) compared to 2020. For this entire cohort, the RTT of ≤ 60 days was achieved higher in 2021 (50.0% *vs.* 8.3%; p=0.019) compared to 2020. Data also showed improvement in RTT of ≤ 60 days for every sequential six months. ‘Non-compliance’ and ‘learning curve’ were the most important factors among patients having the longest RTTs.

Conclusion

The RTT can be further decreased. As a result of this preliminary analysis of the our strategic planning approach of ‘circular’ “See it,” “Own it,” “Solve it,” and “Do it” and go back to the first step again, we plan to implement the following strategies in the immediate future to shorten the RTTs further and, in turn, improve our overall outcomes (local/regional control, disease-free survival, and overall survival): (a) Interdigitate MRI-guided brachytherapy during external beam radiotherapy (EBRT); patients who can not get the interdigitated brachytherapy procedures performed during the course of EBRT for any reason will receive two brachytherapy procedures per week; (c) attempt to add a cervix cancer care navigator to our staff to help patients having social issues, thus leading to compliance problems; (d) finally, in a year or two after these new strategic implementations, the RTT data will be reanalyzed.

## Introduction

Accountability theory

Improvement of systems often requires a multitude of variables to change semi-simultaneously for the improvement strategy to be effective. One effective strategy for accountability, coined “the Oz Principle,” involves four steps to evaluate and improve a system or process: “See it,” “Own it,” “Solve it,” and “Do it” [1]. First, an opportunity must be seen and defined. Secondly, the responsibility must be admitted and goals set for what improvement looks like. Third, distinctive strategy must be developed for how the improvement will happen, which may include many types of “interventions,” such as acquisition of resources, culture change, expansion of capability, etc. Technology may be included, but human factors (leadership, accountability, motivation, humility, etc.) cannot be ignored. Finally, the strategy must be implemented with perseverance for some amount of time, at which point the cycle can be re-initiated should the first iteration prove less effective than desired. This work describes one of our many efforts to implement this accountability strategy into practice in the clinical environment within a radiation oncology department and elucidates how we “saw it,” “owned it,” “solved it,” and “did it” in relation to cervical cancer care.

The opportunity

Evidence is now emerging that the total package time (TPT), meaning the time from the date of diagnosis (usually the date of a biopsy to diagnose a cancer) to the date of completion of all planned treatments, is an important parameter in determining the outcomes (overall survival, disease free survival, local-regional disease control rates, rates of subsequent development of distant metastases) in many cancer sites [2]. In many cancers, a component of the TPT is radiotherapy treatment time (RTT), typically measured from the day of initiation of radiotherapy (RT) to the day of its completion. In the treatment of carcinoma of the uterine cervix (CCx), RTT has been shown to be an important determinant of ultimate outcomes, even when other factors, for instance, stage of the disease, volume of the tumor, presence of co-morbidities, total dose delivered, and adequacy of the RT dose delivered to different target volumes, are all controlled [3].

In the early part of 2020, the department of Radiation Oncology at the University of Mississippi Medical Center (UMMC), undertook a project to decrease the RTT among cervix cancer patients, especially from initiation external beam to complete both external beam and brachytherapy. The hypothesis is that the RTT can be improved if we understood better the causes that led to a delay in the timely completion of the RT among our patient cohort and took remedies to address each or as many of those issues as possible. UMMC is the only tertiary and specialist academic medical center in the state of Mississippi. It also serves as a safety-net medical facility for the state of Mississippi and several proximal cities beyond its borders. Mississippi, however, is a relatively poor and rural state. With this background, we also hypothesized that the hurdles some of our patients face to get an optimal, multidisciplinary, patient focused, evidence based and comparatively prompt diagnosis, followed by treatment for cancer would likely be unique. Hence, the measures to overcome those hurdles must also be distinctive. 

Patients with squamous cell carcinoma of the head and neck (SCCHN) have similar RTT related outcome issues. The survival and local control outcomes worsen in SCCHN when TPT is prolonged [2,4]. Interestingly, both CCx and SCCHN in advanced stages respond well to radiochemotherapy consisting of RT plus generally cisplatin-based chemotherapy. Some of the oropharyngeal SCCHN are also human papilloma virus infection related cancers. We looked at how to improve the TPT in SCCHN [2]. We applied similar strategies in CCx and this report here is our initial analysis of our findings over a period approximately two years. Our intents were to identify the causes specifically of RTT delays and formulate solutions and remedies that could potentially overcome them. Our goals are to continue to monitor these findings with the aim to achieve a less-than-60-day RTT, to maintain those rates, and present our findings in the public domain so that other institutions facing similar issues may benefit from our experience.

When we initiated the project, we classified the potential causes of RTT delays as patient/tumor related factors and system related factors. These potential factors are: age, performance status, insurance status, stage of CCx, transportation issues to make it to the daily external beam RT and magnetic resonance imaging (MRI)-based brachytherapy and having/not having RT specific software (RT specific electronic medical records {EMR} with added features for altering RTT delays).

Whereas it would be virtually impossible for us to change or positively influence the individual patient/tumor related factors, system related factors, however, could potentially be addressed and improved. As our first steps, the department invested in initiating measures to systematically re-organize the logistics of MRI-guided brachytherapy to improve its process efficiency through close collaborations with diagnostic radiology and anesthesiology colleagues; obtain the most current treatment planning software (RayStation Treatment Planning System, Stockholm, Sweden) and EMR (SmartClinic; Elekta, Stockholm, Sweden) systems. Our goal was and is to continue to achieve a ≥ 80% compliance rate in regard to achieving < 60 days RTT, based on the data available in the literature [5,6].

## Materials and methods

Study design and participants

**Figure 1:**
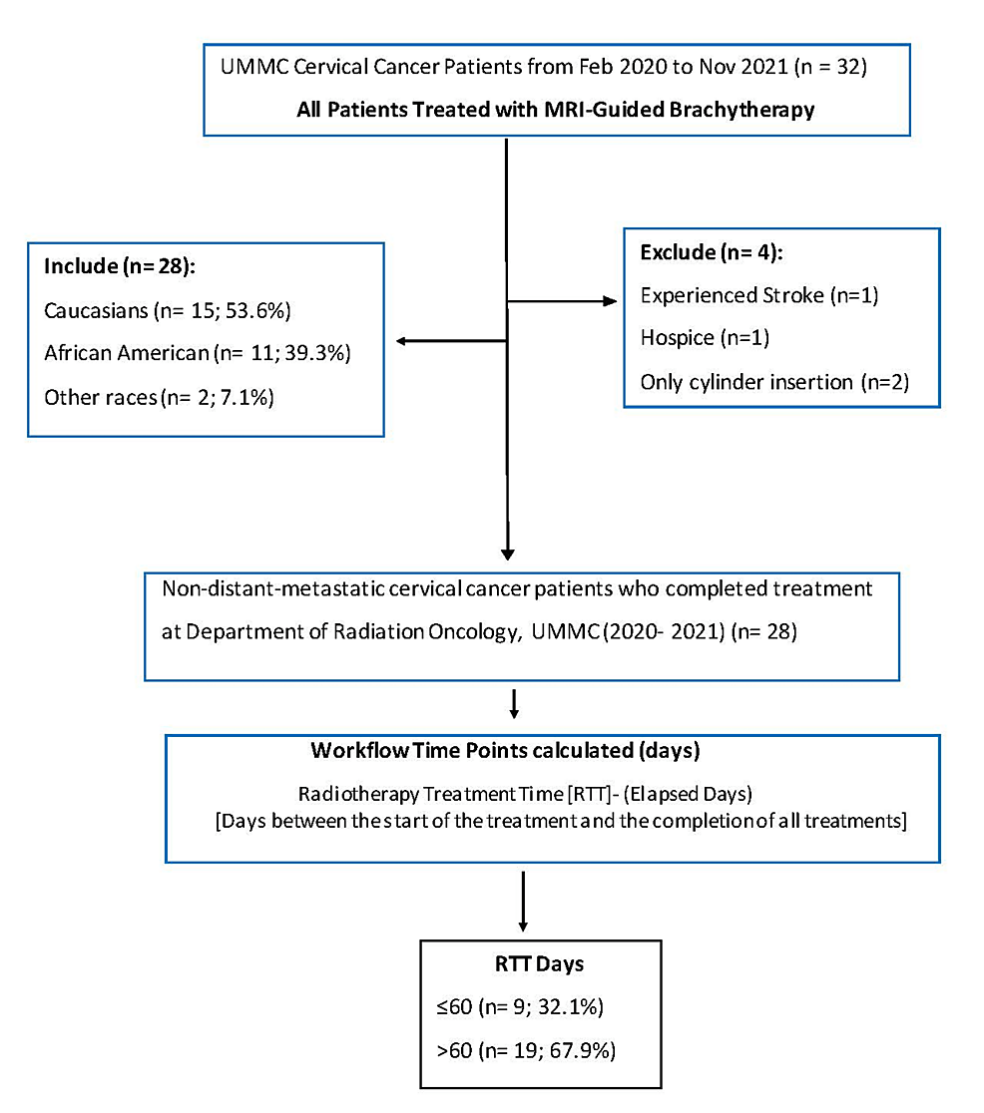
Flow chart for cervical cancer patient cohort selection UMMC: University of Mississippi Medical Center, MRI: magnetic resonance imaging, RTT: radiotherapy treatment time, n: number, %: percentage

This retrospective study includes 28 uterine cervix, non-distant metastatic, cancer patients who were all (except two patients; see section magnetic resonance imaging-guided brachytherapy) treated with MRI-guided brachytherapy at UMMC, Jackson, MS, between February 2020 and November 2021. The UMMC institutional review board approval (IRB # 2012-0147) was obtained and a browser-based database tool, research electronic data capture (REDCap) was used to gather and store patients’ information in password-protected computers. The written consent requirement was waived secondary to the retrospective nature of the study and all patient identifiers were removed before the data was extracted. Of the 32 patients treated during this period, four patients who did not or were unable to complete the planned treatment were excluded. The first patient had a hemorrhagic stroke after the first high-dose radiation (HDR) insertion (on the same night as the first insertion, and this was deemed not to be due to the brachytherapy); patient number two became ill and elected hospice care; patients three and four were both postoperative patients who were treated with only vaginal cylinder insertion. The remaining 28 cervical cancer patients were included in this analysis (shown in Figure 1).

Data collection

Epidemiological, clinical, demographic, and treatment data was obtained from REDCap. The patients were all stratified by RTT, the number of days between the start of the RT and completion of the treatment (≤ 60 *vs.* > 60 days). The following patient characteristics were also included: race/ethnicity, age, distance travelled to the facility, income level, insurance status, and Federation Internationale de Gynecologie et d'Obstetrique (FIGO) stage of disease. All data were collected, checked, analyzed, and interpreted by the research faculty (MN) and verified by co-authors. 

Definitions

RTT: We defined RTT as the time from the date of initiation of external beam radiation treatment (EBRT) to the completion of all radiation treatments.

Strategic planning*:* Following the ‘Accountability theory’ and ‘Oz Principle’ of “See it,” “Own it,” “Solve it,” and “Do it”, specific attention was paid to decrease the RTT [2].

Issues*: *Non-compliance - reluctance/inability to report for daily EBRT/ brachytherapy despite repeated counseling, phone calls, and reminders. Learning curve - earlier part of the MRI-guided brachytherapy, especially the interstitial implants. Stage IV/comorbidities - patients with stage IV disease with tumor-induced ‘comorbidities’ such as nephrostomies, extensive bleeding, venous thrombosis, failure to thrive, etc. More than one issue - meaning more than one of the listed three issues. Our hypothesis was that these issue(s) would lead to increased RTT.

Treatment details

External Beam Radiation Therapy Planning

External beam was administered to each patient to a total dose between 45 to 50.4 Gy in 25 to 28 fractions, respectively. Initially, patients from early 2020 were treated with either 3-dimensional (3D) or intensity modulated radiation therapy (IMRT)/volumetric modulated arc therapy (VMAT) techniques at the attending physician’s discretion, but departmental preference moved towards the more complex and departmental peer-review tumor board preferred IMRT/VMAT technique for more recent patients treated in 2021 [7]. All patients underwent computed tomography (CT) simulation which was then merged with the diagnostic MRI and positron emission tomography-computed tomography (PET-CT) imaging prior to treatment planning. External beam plan design and calculations were performed initially with the pinnacle treatment planning system (Philips Medical Systems, Fitchburg, WI); however, the department transitioned to RayStation (Raysearch Laboratories, Stockholm, Sweden) during the study period. Treatment was delivered with daily treatment fractions of 1.8 to 2 Gy, excluding weekends and institution holidays. Brachytherapy was either interdigitated with the external beam treatments or initiated at the conclusion of the external beam portion of the treatment course. 

Chemotherapy

For a majority of patients, cisplatin based weekly chemotherapy concurrently with RT is the standard [8]. For those patients not tolerating cisplatin chemotherapy, carboplatin can be a good substitute [8]; an alternative regimen among those patients intolerant of cisplatin is a combination of cisplatin plus fluorouracil given every three to four weeks can be considered [8,9]. The role of a combination of cisplatin plus gemcitabine is still evolving [10,11]. The role of targeted therapy and precision therapy/radiobiotherapy in the future needs to be defined and likely to improve the outcomes [12,13]. 

Our treatment regimen in the Division of Gynecologic Oncology at UMMC follows the standard National Comprehensive Cancer Network (NCCN) guidelines of concurrent chemoradiotherapy with weekly cisplatin at a dose of 40 mg/m^2^ during primary EBRT for targeted 5-6 weeks. Acute side effects and toxicity are monitored and managed both inpatient and outpatient in attempts to stay on therapy without delay. If grade 3-4 toxicities persist, discontinuation of weekly cisplatin may be implemented. If cisplatin is poorly tolerated in the first two cycles administered, substitution with carboplatin has been used.

Magnetic Resonance Imaging Guided Brachytherapy

All patients reported upon in this study underwent MRI-guided high-dose-rate (HDR) brachytherapy, typically in four fractions, with each dose of 7 Gy delivered weekly [14]. We are now planning to move to two insertions per week, Mondays and Fridays to shorten the RTT further. Every patient underwent MRI imaging with the applicator in place for the first fraction and the high-risk clinical target volume (HRCTV) was delineated per American Brachytherapy Society (ABS) guidelines [15,16]. When logistical issues arose due to both patient volume and MRI availability, future fractions could have used CT only fused with the initial MRI registration if the volume was small and confined to the cervix. MRI was generally utilized for all HDR treatment fractions for each patient except for two whose disease was localized to the cervix only; for these two, subsequent treatments after the first were performed with only CT after image fusion with the first fraction MRI due to high patient volume and low MRI availability. All other patients had four MRI- based brachytherapy treatments. Nonetheless, the HRCTV was re-delineated for each treatment fraction based on the current anatomy for that day. Diagnostic radiology consultation was utilized, at least, for HRCTV delineation in the first fraction. The initial treatment planning goal was always to achieve a dose received by 90% of the cervix (D90) of at least 7 Gy; however, the American Society for Radiation Oncology (ASTRO) consensus goals of 80 to 85 Gy equivalent total doses in 2 Gy fractions (EQD2) guided the desired fractional D90 dose. For a subset of patients, when the ideal D90 was not achievable due to HRCTV size/shape or normal tissue concerns, interstitial needles were added to the remaining fractions using the venezia applicator (Elekta, Stockholm, Sweden). An equivalent dose (EQD2) of at least 80 Gy was achieved when volumetrically feasible, falling back on traditional point A prescription only when the intent became palliative due to disease extent (the same two patients mentioned above who were prescribed to the point A). The technical aspects of the MRI-guided procedure are very similar to those described by Zhang et al. [17].

Patient management

**Figure 2:**
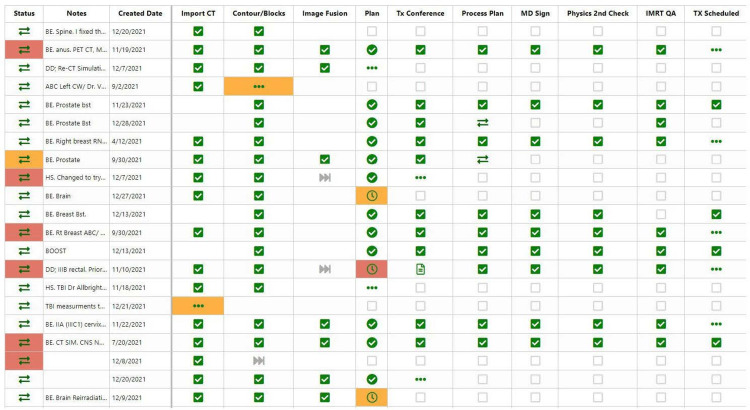
Screenshot of a portion of the “simulation to treatment” SmartBoard CT: computed tomography, IMRT: intensity modulated radiation therapy, Tx: treatment, QA: quality assurance, BE, DD, and HS: planner initials, ABC: active breathing coordinator, TBI: total body irradiation, SIM: simulation, Orange color coding: task reached its due date, Red color coding: task has past its due date Note: similar software are available through other vendors ARIA Care Paths [18]

As of March 2021, SmartClinic SmartBoards (Elekta, Stockholm, Sweden) was online and utilized to enhance our mosaiq “record-and-verify” system for patient management (shown in Figure 2). 

After the initial consultation, the patient enters the SmartBoard radar screen and is subsequently monitored to ensure that preparatory work for the CT simulation is completed. Once CT simulation is completed, the patient enters a more rigorous workflow where every step is individually monitored to convey the patient from CT simulation to their first EBRT as quickly as possible. Steps are color-coded based on whether departmental deadlines are being met and SmartBoards are reviewed regularly during peer-review tumor boards to ensure timely patient management [19,20]. Once the patients are out of the external beam planning process, they remain on the attending’s scheduling for HDR brachytherapy treatment management.

Statistical analyses

For the normally distributed data, the categorical variables were presented by absolute numbers and percentages. The relationship between independent variables, including sociodemographic factors, type of insurance, type of issues present, FIGO staging, and the dependent variable RTT, were analyzed using simple linear regression. The respective p-values were recorded, and p values ≤ 0.05 were considered statistically significant. Box plots were also generated to compare the data distribution between groups. Data were analyzed using statistical package for the social sciences (SPSS) software, version 28.0 (IBM, Armonk, NY, USA).

## Results

Thirty-two cervical cancer patients were treated at UMMC between February 2020 and November 2021. Four of these patients were not included in the analysis as reported above (Figure 1). The balance of 28 patients who were treated with MRI guided brachytherapy met the final inclusion criteria. The baseline characteristics of these patients (Table 1) described a median age of 49 years (range, 28 to 77 years) and a higher percentage of Caucasians to African American and other races (53.6%, 39.3 %, and 7.1%) during the time of this study. 

**Table 1:** Descriptive analysis for radiotherapy treatment time of cervical cancer patients (n=28) RTPT: radiotherapy total package time, RTT: radiotherapy treatment time, FIGO: Federation Internationale de Gynecologie et d'Obstetrigue, n: number, %: percentage

Variable	Median RTPT	RTPT ≤ 60 (n=9)	RTPT > 60 (n=19)
Race			
Caucasian	63.0	6 (66.7%)	9 (47.4%)
African American	71.0	2 (22.2%)	9 (47.4%)
Other Races	66.5	1 (11.1%)	1 (5.3%)
Age (years)			
≤50	69.5	5 (55.6%)	11 (57.9%)
> 50	64.5	4 (44.4%)	8 (42.1%)
Distance (miles)			
≤60	68.0	3 (33.3%)	6 (31.6%)
> 60	68.0	6 (66.7%)	13 (68.4%)
Income ($)- State of Mississippi poverty cutoff level			
≤ $ 35000	63.0	2 (22.2%)	3 (15.8%)
> 35,000	71.0	7 (77.8%)	16 (84.2%)
Insurance			
Medicaid	71.0	4 (44.4%)	11 (57.9%)
Medicare	73.0	0 (0.0%)	6 (31.6%)
Private	57.0	2 (22.2%)	1 (5.3%)
Self-pay	55.0	3 (33.3%)	1 (5.3%)
Issues			
No issues	57.0	7 (77.8%)	5 (26.3%)
Non-compliance	80.5	0 (0.0%)	8 (42.1%)
Stage IV/ comorbidities	63.0	2 (22.2%)	3 (15.8%)
Learning Curve	92.0	0 (0.0%)	3 (15.8%)
FIGO Stage			
IB	68.0	1 (11.1%)	2 (10.5%)
IIB	61.5	4 (44.4%)	4 (21.1%)
IIC	56.0	1 (11.1%)	0 (0.0%)
IIIB	78.0	0 (0.0%)	3 (15.8%)
IIIC	69.5	1 (11.1%)	7 (36.8%)
IVA	71.0	2 (22.2%)	2 (10.5%)
IVB	80.0	0 (0.0%)	1 (5.3%)

The median RTT was 68 days (range, 51 to 106 days) for the entire cohort. Patients were stratified by RTT intervals (days) between the start of the treatment (the day EBRT began) and the completion of all treatments into two groups (≤ 60 {32.1%} *vs.* > 60 days {67.9%}), (shown in Figure 1). The following patient characteristics were included: race/ethnicity, age, distance travelled for treatment, income level, insurance status, issues causing longer RTT, and FIGO staging.

The majority of patients in RTT ≤ 60 group were Caucasians (66.7%). The type of insurance played a significant role, with the Medicaid patients having more difficulty meeting the ideal RTT of < 60 days. The presence of other issues (non-compliance, stage IV/comorbidities, patients treated during learning curve in the use of MRI-guided brachytherapy (early days of implementation of our MRI-guided brachytherapy program) influenced a delay in completing the prescribed treatment within the RTT in days studied. In other words, “no issues” were documented more in the lower RTT group (77.8%) compared to the higher RTT group (26.3%). Case-by-case details related to comorbidities/medical factors and non-compliance for all 28 patients are listed in Table 2.

**Table 2:** Details of comorbidities/ medical factors and non-compliance in the patient cohort (case-by-case instances) C: Caucasian, AA: African-American, O: others, RTT: radiotherapy treatment time, FIGO: Federation Internationale de Gynecologie et d'Obstetrigue, HDR: high dose rate, MRI: magnetic resonance imaging, RT: radiation therapy, EBRT: external beam radiation therapy, hr.: hour, min: minutes, N/A: not-applicable, UMMC: University of Mississippi Medical Center

Patient number	Age (years)	Race	Insurance	RTT (days)	FIGO stage	Stage IV	Stage IV related comorbidity	Lack of compliance	Reason for non-compliance	Comment	Brachytherapy learning curve
1	58	C	Medicare	85	IIB	No	No	Yes	Not from greater Jackson area - home 1 hr. and 15 min by drive	Received external beam closer to home; No brachytherapy available closer to home. Only brachytherapy at UMMC	No
2	54	C	Private	56	IIC	No	No	No	N/A	N/A	No
3	48	AA	Private	66	IIB	No	No	Yes	Not from greater Jackson area - home 2 hr. and 10 min by drive	Received external beam closer to home; No brachytherapy available closer to home. Only brachytherapy at UMMC	No
4	47	C	Private	57	IIB	No	No	No	N/A	N/A	No
5	40	AA	Medicaid	51	IB	No	No	No	N/A	N/A	No
6	56	C	Medicaid	51	IIB	No	No	No	N/A	N/A	No
7	35	C	Medicaid	59	IVA	Yes	HDR abandoned after two HDRs due to huge tumor volumes (MRI based tumor extension even after external beam was: large cervical mass involving the lower uterus, entire length the cervix, and upper vagina 5.0 x 4.1 x 5.5 cm with parametrial extension resulting in encasement of both ureters and upstream bilateral hydroureter). Patient deceased.	No	N/A	N/A	No
8	44	C	Medicaid	76	IIIC	No	No	Yes	No show for initial consult, no show for initial planned HDR; required multiple phone calls and emails to bring back; not from greater Jackson area- home 2 hr. and 55 min by drive	Received external beam closer to home; No brachytherapy available closer to home. Only brachytherapy at UMMC	No
9	48	C	Medicaid	78	IIIB	No	No	Yes	No show; required multiple phone calls and emails to bring back; not from greater Jackson area - home 1 hr. and 55 min by drive	Received external beam closer to home; No brachytherapy available closer to home. Only brachytherapy at UMMC.	No
10	46	AA	Medicaid	71	IIIC	No	No	No	N/A	N/A	Yes
11	38	AA	Medicaid	68	IB	No	No	No	N/A	N/A	No
12	59	AA	Medicaid	71	IB	No	No	No	N/A	N/A	No
13	40	AA	Medicaid	92	IIB	No	No	No	N/A	N/A	Yes
14	60	C	Medicaid	83	IVA	Yes	Weight loss of 135 lbs. when presented for treatment, delayed diagnosis due to husband's illness and death, needed fixation of right distal radius fracture with surgical intervention during the course of RT, requiring treatment break, EBRT 25 fractions in 49 days due to surgery for fracture as well as RT being on hold to hospital admission from Cisplatin related complications of nausea, vomiting and diarrhea	Yes	Not from greater Jackson area - home 1 hr. and 10 min by drive	Both external beam RT and brachytherapy at UMMC.	No
15	49	C	Medicaid	51	IVA	Yes	Cardiomegaly	No	N/A	N/A	No
16	31	C	Medicaid	68	IIIC	No	No	Yes	Not from greater Jackson area - home 1 hr. and 25 min by drive	Received external beam closer to home; No brachytherapy available closer to home. Only brachytherapy at UMMC.	No
17	50	AA	Medicaid	72	IIIC	No	No	No	N/A	N/A	No
18	40	AA	Medicaid	98	IIIC	No	No	Yes	Not from greater Jackson area - home 1 hr. and 25 min by drive	Received external beam closer to home; No brachytherapy available closer to home. Only brachytherapy at UMMC.	No
19	36	C	Medicaid	94	IVA	Yes	Severe right sided and mild left-sided hydroureteronephrosis with obstructive uropathy	No	N/A	N/A	No
20	75	C	Medicare	63	IIIC	No	No	No	N/A	N/A	No
21	67	C	Medicare	66	IIIB	No	No	No	N/A	N/A	No
22	28	AA	Medicare	91	IIIB	No	No	Yes	No show multiple appointments; required multiple phone calls and emails to bring back; not from greater Jackson area- home 1 hr. and 55 min by drive	Received external beam closer to home; No brachytherapy available closer to home. Only brachytherapy at UMMC.	No
23	49	O	Medicare	80	IVB	Yes	Pyometria, coronary heart disease	No	N/A	N/A	No
24	53	O	Self-pay	53	IIB	No	No	No	N/A	N/A	No
25	56	AA	Self-pay	106	IIB	No	No	No	N/A	N/A	Yes
26	50	C	Self-pay	57	IIB	No	No	No	N/A	N/A	No
27	45	AA	Self-pay	51	IIIC	No	No	No	N/A	N/A	No
28	77	C	Medicare	62	IIIC	No	No	No	N/A	N/A	No

The simple linear regression is a parametric test used to estimate the relationship between one independent and one dependent variable using a straight line. Based on Table 3, the issue category variables non-compliance (p=0.001) and learning curve (p<0.001) showed a positive significant linear relationship towards shorter RTT.

**Table 3:** Simple linear regression CI: confidence interval, FIGO: Federation Internationale de Gynecologie et d'Obstetrigue

Variable	β (95% CI)	p- value
Race		
Caucasian	1	
African American	0.285 (-3.80 - 21.85)	0.160
Other Race	-0.009 (-24.90 - 23.77)	0.962
Age (years)		
≤ 50	1	
> 50	-0.102 (-15.73 -9.36)	0.606
Distance (miles)		
≤ 60	1	
> 60	0.025 (-12.51- 14.20)	0.898
Income ($)- State of Mississippi poverty cutoff level		
≤ $ 35000	1	
> 35,000	0.059 (-13.86 - 18.66)	0.764
Insurance		
Medicaid	1	
Medicare	0.061 (-13.67 - 18.27)	0.769
Private	-0.250 (-33.45 - 8.38)	0.228
Self-pay	-0.123 (-24.06 - 13.16)	0.551
Issues		
No issues	1	
Non-compliance	0.545 (9.00 - 31.74)	0.001
Stage IV/ comorbidities	0.187 (-5.70 - 20.80)	0.124
Learning Curve	0.588 (13.34 - 45.49)	<0.001
FIGO Stage		
IB	1	
IIB	0.220 (-16.38 - 31.47)	0.519
IIC	-0.088 (-48.14 - 33.47)	0.712
IIIB	0.300 (-13.85 - 43.85)	0.292
IIIC	0.198 (-17.13 - 30.72)	0.561
IVA	0.190 (-18.57 - 35.41)	0.524
IVB	0.200 (-24.14 - 57.47)	0.405

The overall percentage of the RTT interval group of 60 days is shown (≤ 60 {32.1%} *vs.* > 60 {67.9%}). For this entire cohort, the presence of three factors/issues were analyzed: inability to attend regularly (non-compliance {≤ 60 (0.0%} *vs. *> 60 days {100.0%}); presence of stage IV/co-morbidities (≤ 60 {40.0%} *vs.* > 60 days {60.0%}; patients treated during the learning curve in the use of MRI-guided brachytherapy (≤ 60 {0.0%} *vs. *> 60 days {100.0%}); and none of these factors/issues being present (≤ 60 {58.3%} *vs.* > 60 days {41.7%}) (Table 4).

**Table 4:** Breakout of the patient population in terms of percentages with ideal RTT intervals RTT: radiotherapy treatment time, %: percentage

Patient population	≤ 60 days	> 60 days
Overall percentage for the entire population	32.1%	67.9%
For the population with no issues	58.3%	41.7%
For the population with non-compliance	0.0%	100.0%
For the population with learning curve	0.0%	100.0%
For the population with stage IV/comorbidities	40.0%	60.0%
For the population with more than one issue	12.5%	87.5%

**Figure 3:**
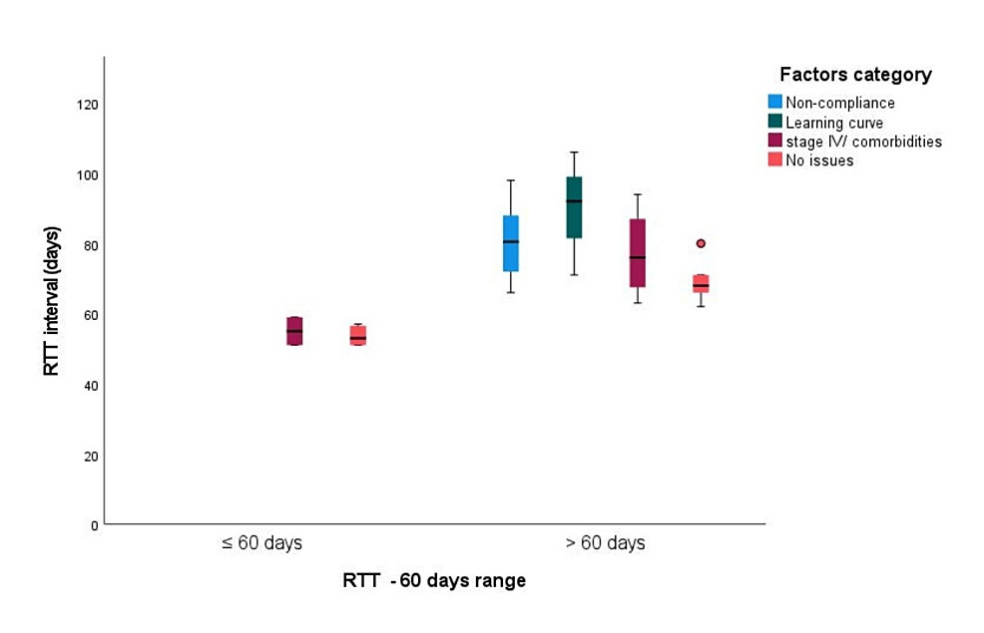
Box-Whisker plot for radiotherapy treatment time (60 days goal) – overall factors influencing the radiotherapy treatment time durations RTT: radiotherapy treatment time; note that the < 60 days group has a 'narrower' spread of RTT in days compared to the > 60 days group; orange circle: is an outlier - the data point numerically distant from the rest of the data

The presence of one or more factors led to a prolonged RTT compared to the absence of those three (shown in Figure 3).

**Figure 4:**
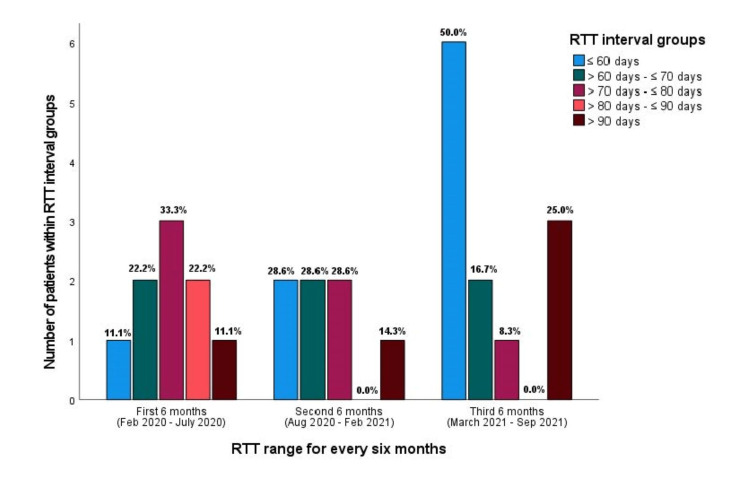
Radiotherapy treatment time comparisons for every six-month interval during the entire study period RTT: radiotherapy treatment time, %: percentage

Figure 4 shows the RTT comparison for each six-month interval during the entire study period. The data show a trending improvement in decreased RTT at the latter periods, likely due to the measures implemented (in the third six-month period RTT ≤ 60 days was 50.0% compared to 11.1% during first six month-period), although the p-values were insignificant due to the small patient numbers under individual categories.

The RTT of ≤ 60 days was achieved in 50% of patients in 2021 compared to only 8.3% of patients in 2020 with a p-value of 0.019 (Table 5). 

**Table 5:** Descriptive statistics for radiotherapy treatment time for cervical cancer patients RTT: radiotherapy treatment time, n: number, %: percentage

RTT (calendar year)	≤ 60 days	> 60 days	p-value
2020 (n=12)	1 (8.3%)	11 (91.7%)	0.019
2021 (n=16)	8 (50.0%)	8 (50.0%)	

The three dimensional conformal radiotherapy (3D-CRT) and IMRT are two techniques of radiotherapy used in the RT of cervix cancer. IMRT is believed to have fewer side effects than 3D-CRT [21]. In this study population, the majority of the patients (87.5%) in 2021 underwent treatment using IMRT compared to 8.3% in 2020 with a significant p-value of <0.001 (Table 6).

**Table 6:** The distribution of the type of external beam radiation therapy stratified by the calendar years 3D- CRT: three dimensional conformal radiation therapy, IMRT: intensity-modulated radiation therapy, n: number, %: percentage

Calendar years	3D- CRT	IMRT	p-value
2020 (n=12)	11 (91.7%)	1 (8.3%)	<0.001
2021 (n=16)	2 (12.5%)	14 (87.5%)	

Twenty-one patients underwent intracavitary and seven interstitial brachytherapy. The RTT of ≤ 60 days was achieved in 28.6% of intracavitary patients compared to 42.9% of patients who underwent interstitial brachytherapy with no significant p-value.

Table 7 demonstrates the analysis results with the breakdown of the “no Issues” factor by calendar year. The RTT of ≤ 60 days was achieved in 85.7% of patients in 2021 compared to 20.0% of patients in 2020 with a significant p-value of 0.023.

**Table 7:** Descriptive statistics for radiotherapy treatment time of 'no issues' per calendar year RTT: radiotherapy treatment time, n: number, %: percentage

RTT (calendar year)	≤ 60 days	> 60 days	p-value
2020 (n=5)	1 (20.0%)	4 (80.0%)	0.023
2021 (n=7)	6 (85.7%)	1 (14.3%)	

## Discussion

As early as 1993, the patterns of care (POC) study of cervix cancer in the USA showed the adverse influence of prolonged RTT on survival (p=0.001) and pelvic disease control (p=0.001) among 837 stages I-III patients treated with RT alone [22]. The other independent factors that adversely affected the outcomes were age (less than versus more than 50 years) and stage of the disease (I *vs.* II *vs.* III). An earlier POC study demonstrated that elderly patients, patients with higher karnofsky performance status, and unilateral parametrial involvement in stage IIB/unilateral side wall involvement in stage III had better outcomes [23]. Similar single institution experience was reported by Perez et al., from Washington University among 1,224 patients, again, in pre-chemoradiotherapy era. A 10-year pelvic failure was found in 7% with < seven weeks of RTT versus 22% with 7.1 to nine weeks RTT versus 36% with > nine weeks (p = 0.01 among stage IB patients). Similar influence of RTT was found for pelvic disease control and caused specific survival for other stages as well (stages IB - III) [24].

There are data pointing to the similar importance of RTT in the chemoradiotherapy (chemoRT) era as well. For example, Song et al., emphasized the importance of keeping RTT to within eight weeks from a single institution experience [25]. In a multi-institutional pooled analysis involving centers in Europe, United Kingdom, and USA, Tanderup et al., emphasized the importance of diminished RTT in a state of the art chemoRT intervention that included MRI-guided brachytherapy [26]. An HRCTVHR (D90-high risk clinical target volume) dose of > 85 Gy delivered over seven weeks provided the best outcomes in this study. Our approach to treatment interventions are similar to the one reported in this study with chemoRT combined with MRI-guided brachytherapy. French clinical oncologists from Gustave Roussy Cancer campus reported similar findings among 225 patients with modern chemoRT combined with image-guided adaptive brachytherapy similar to our department’s approach in treating cancer of the uterine cervix [27]. In addition to the individual and multi-institutional data summarized above, the National cancer database (that includes not only academic centers but also non-academic cancer care facilities) analyses also point out the importance of RTT. Hong et al. performed a recursive partition analysis with bootstrapped aggregation (bagging) among 7,355 patients treated between 2004 and 2012 with chemoRT, including brachytherapy (although this was before the MRI-guided brachytherapy era), and reported that overall survival deteriorated if the RTT was more than 64 days [28].

Although at UMMC, we are able to provide state-of-the-art multidisciplinary treatment for cervix cancer that incorporates IMRT/IGRT, concurrent chemotherapy, and MRI-guided brachytherapy, we were concerned that the social determinants of disease would influence the RTT and in turn the patient outcomes. Our concerns are proven correct from our analysis of two years of data, especially that from the first year of the study. A recent evidence-based review by the Society of Gynecologic Oncology (SGO) on the influence of health inequities/social determinants of disease on outcomes in gynecologic cancers indicates black race, low English fluency, and rural residency all have an adverse impact on the outcomes of gynecologic cancer treatment, including the cancer of the cervix [29]. This report pointed out that the influence of the disparities in the outcomes are multifactorial, the interaction between social determinants of health, disparities and health systems are complex, it is not easy to measure the disparities accurately, and multilevel interventions are required to achieve equity in providing an optimal care and attaining comparable outcomes [29]. Our results show a higher percentage of patients having > 60 days median RTT among African Americans, ≤ $ 35,000 annual income group and Medicare/Medicaid insurance patients may represent surrogate measures of heath inequities/unfavorable social determinants of disease.

We analyzed three factors/issues that influence the RTT duration: a) patients with advanced stage IV disease/comorbidities; b) patients who had difficulty with compliance of the rigorous daily treatment requirements during EBRT or with brachytherapy; c) during the first year of our MRI based brachytherapy learning curve; and d) a combination of one or more of those three items. These findings prove our previous hypothesis [4] that technology alone cannot solve the socio-demographic issues. A human touch is always needed [30]. The rest of the discussion will focus on this ' human- (touch)-factor'.

As possible solutions, the SGO report [29] recommended the following for gynecologic cancers including cervix cancer: (1) Incorporation of patient navigation: Such a cancer care navigator will provide a patient-centered barriers overcoming healthcare delivery assistance that is beyond just helping with community outreach. Cancer care circa 2022 is complex, multidisciplinary, multilayered, highly specialized, and volume-based-competency-based. Our experience, qualitatively and quantitatively, unfortunately confirms the findings of SGO. Despite our best efforts with strategic planning, investment into modern technology (SmartClinic, RayStation, and MRI-guided brachytherapy), leadership commitment, prospective education to the patients and support staff, our improvements in RTT in cervix cancer have not yet achieved level we aimed for. We hypothesize that a navigational support person could help us climb the final “few steps” to reach the goal. (2) Health Care System Improvements: The details of these essential efforts are beyond the scope of this report.

Since the improvement in the outcomes of cervix cancer was achieved with the introduction of chemoradiotherapy, four major advances in the radiotherapeutic management of non-distant-metastatic cervix cancer have occurred. These are a) the recognition of the importance of staging using PET/CT and its use in external RT planning [31], b) the use of IMRT in the EBRT improving the therapeutic ratio, c) the use of MRI-based brachytherapy [13], and d) finally, the observation that the RTT is still important despite the other improvements listed above. The last item was well documented in the recent paper from the University of Pittsburgh Medical Center’s retrospective analysis that looked at the importance of high HRCTV volumes in influencing poorer outcomes despite delivering higher doses with brachytherapy, that became possible with the use of MRI-based brachytherapy [32]. In the univariate analysis, the factors that emerged to be important in the control of local disease were adenocarcinoma histopathology, dose prescription at the time of brachytherapy, the HRCTV volume and the RTT (of less than versus more than 51 days). For distant metastasis likelihood, the factors were lymph-nodal positivity status, use of a hybrid applicator enabling interstitial implant during brachytherapy, RTT and HRCTV. For overall survival, however, the factors were age, clinical tumor size, RTT, HRCTV, and HRCTV-D90 dose more than 81 Gy [32]. It is noteworthy that RTT was important in all those outcomes studied; in the multivariate analyses also RTT remained significant in two of those three parameters mentioned, distant metastasis rates and the likelihood of overall survival. It appears that RTT is still relevant despite many innovations that have occurred and have improved the overall outcomes of the cervix cancer.

In similar efforts in SCCHN, we used radiotherapy total package time (RTPT), typically measured from the day of consult for RT to the day of its completion, to show similar improvements using similar strategies [2]. The demonstration in two different disease sites with similar strategies suggests systematic planning and execution of efforts can lead to improvements in avoiding delays in cancer care operational executions. It must be clearly understood that TPT has many components: (a) The time from a patient’s recognition of something is wrong to show up at the doors of a care provider - ‘Symptom to Action Time'. This probably is the most difficult to measure and the one likely to have most variations between different disease sites and geographical locations. (b) The time from first care provider interaction to pathological diagnosis of cancer - 'First Interaction to Diagnosis Time' (c) The time from pathological diagnosis to the first treatment initiation - 'Diagnosis to Treatment Initiation Time' (d) And finally, the time from the first day of first treatment to completion of all treatments - 'First Treatment to Treatment Completion Time'. Similarly, RTPT has many components [2]. Each one of these time intervals is important and need to be shortened to get the best possible outcomes and will need different strategies.

Since this is not our only effort at the application of accountability theory to our practice, we can compare the “lessons” we have learned from both efforts. In addition to decreasing package time for CCx patients, we also applied this approach to decreasing RTPT for our head and neck cancer patients as well. Due to meaningful improvement in this area as well (mean and median reductions in RTPT of almost 23 days and 14 days, respectively), we can draw out common themes from both efforts [2]. Firstly, improvement can often be realized when attention and effort is applied whether using this accountability theory or another. While not all things measured matter, in both areas here, TPT, which may be critical to outcomes, not only was measured, but was affected by strategic efforts. Secondly, technology can have a large role in offering advancement in the clinic. Since both efforts involved the application of information and communication technology (ICT) in the form of SmartClinic, the expansion of the abilities of our human practitioners is noted [4]. ICT seems likely to play a large role in our clinical management of patients for the future. Thirdly, without addressing human factors through leadership, motivation, and culture change, any technological intervention may be largely limited in impact. When addressing hardware or software, often maintenance or a line of code may improve performance, but with humans, positive impact requires many factors such as efficacious positive influence and humility towards personal development. Additionally, despite our significant improvements in both areas, the opportunity for further improvement remains. For example, neither of our strategies for each area fully addressed all patient-related factors, which could be socioeconomic or geographic, affecting patient attendance to planned appointments. Finally, while parts of the strategy for both areas of improvement looked quite different, often portions of the strategy for one opportunity may be a significant contributor in other areas as well (i.e. implementation of ICT).

**Figure 5:**
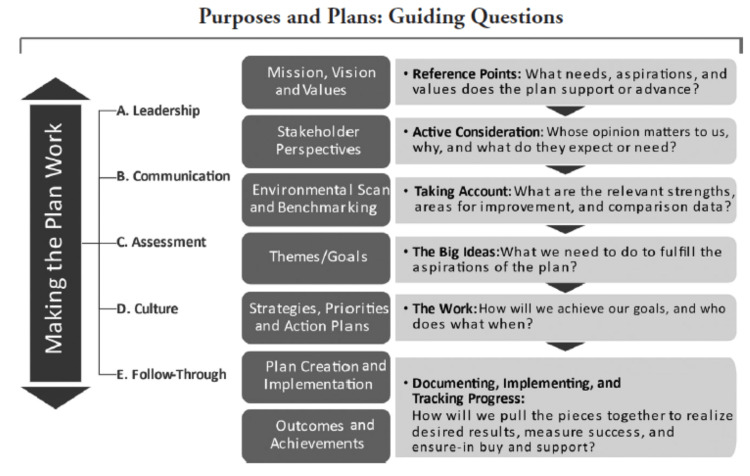
Strategic planning in higher education This image is reproduced from Ruben et al. [34]. Permission to reproduce was obtained from the licensed content publisher Stylus Publishing.

The importance of ‘strategic planning’ in accomplishing goals in a clinical medicine department is well illustrated by our efforts, both in head and neck cancer and cervix cancer, in decreasing the RTPT and RTT, respectively. Dweik et al., from their recent paper for chest clinicians about strategic planning, posit that: (a) having a strategic vision, (b) a strategic plan, and (c) an organizational culture that is healthy and vibrant are all important to achieve high organizational performance and success [33]. This is consistent with other sources that emphasize creating, organizing, planning, implementing, and monitoring successful strategic planning. Such a plan needs to follow the pathways suggested in figure 5 [34].

We followed those steps in our strategic planning and execution to decrease the RTPT/RTT. Table 7 below shows further detail of our approach specific to this project [33].

**Table 8:** Steps in developing strategic planning for our RTPT/RTT project RTPT: radiotherapy total package time, RTT: radiotherapy treatment time The left column is reproduced from Dweik et al. [33]. Permission to reproduce was obtained from the licensed content publisher Elsevier. The right column is our approach for this specific project.

Steps of strategic planning		Actions to decrease the RTPT/RTT
1	Determine who the customer is	Our patients
2	Develop a vision	To decrease RTPT/RTT, as much as possible
3	Recruit a guiding coalition to develop a plan to implement the strategy. Optimize teamwork among this guiding coalition	The whole department, with the operational coalition being the physicians, physicists, and dosimetrists
4	Articulate the vision to the members of the guiding coalition and establish their roles, goals, and responsibilities	This was articulated in the initial stages in department wide conferences and then reinforced during daily huddles and peer review tumor boards
5	The guiding condition presents the plan to leadership and hopefully receives endorsement; alternatively the plan is iterated to a finally endorsed plan	The leadership of the department, in fact, was part of the planning team and hence there was full enforcement and support from day 1
6	Implementation begins, measuring outcomes along the way with a Plan-Do- Check -Act-Approach	The current reports are part of this process of measuring the outcomes: 'The Check-Act' cycle
7	Continue measuring outcomes to ascertain success. Once achieved, celebrate success	These will be our next steps: where do we have to focus? How to improve further? Celebrate the current success

Our success shows that strategic plans help succeed in clinical medicine departments, not just for big corporations and huge academic medical centers [35]. Of further importance to us is that strategic planning allowed us to consider and include our perceived strengths, such as prospective peer review of such patients, rather than ignoring or even detracting from progress made in those areas [19,20,36,37] .

## Conclusions

We plan to implement the following strategies in the immediate future to shorten the RTTs and, in turn, improve our overall outcomes (local/regional control, disease-free survival, and overall survival): (a) Interdigitate MRI-guided brachytherapy during EBRT among patients who are not referred to us after EBRT for brachytherapy only; (b) the latter group of patients and those who could not get all their brachytherapy procedures performed during the course of EBRT will receive two brachytherapy procedure per week, thus shortening the RTT by two weeks; (c) attempt to add a cervix cancer care navigator to our staff to help patients having social issues, thus leading to compliance problems; (d) finally, as part of our strategic planning and determination to follow the accountability theory, in a year or two after these new strategic implementations, the RTT data will be reanalyzed.

In an academic state institution in a rural, resource scarce state, it is demonstrated that careful strategic planning and focus on operational details can improve RTT in CCx. These efforts did not require extensive expense nor huge infrastructure investment. This report demonstrates that careful creative strategic planning without enormous investment can lead to improvements in cancer care and can help other countries, provinces/states, and regions with similar rural and/or resource lean environments. It is also demonstrated that technical improvements alone are unlikely to yield the best results; there is a clear need for human interventions.

The efforts to improve not only RTT but also the other components of TPT/RTPT will be important and need to be actively pursued to improve the outcomes in cancer care.
